# Contemporary temperature-driven divergence in a Nordic freshwater fish under conditions commonly thought to hinder adaptation

**DOI:** 10.1186/1471-2148-10-350

**Published:** 2010-11-11

**Authors:** Kathryn D Kavanagh, Thrond O Haugen, Finn Gregersen, Jukka Jernvall, L Asbjørn Vøllestad

**Affiliations:** 1 Evolution and Development Unit, Institute of Biotechnology, University of Helsinki, P.O. Box 56 (Viikinkaari 9), 00014 Helsinki, Finland; 2 Norwegian Institute for Water Research, Gaustadalléen 21, NO-0349 Oslo, Norway; 3 Centre for Ecological and Evolutionary Synthesis, Department of Biology, University of Oslo, P. O. Box 1066 Blindern, NO-0316 Oslo, Norway; 4 Hedmark University College, Campus Evenstad, NO-2418 Elverum, Norway; 5 Department of Ecology and Evolution, Stony Brook University, Stony Brook, NY, USA; 6 Department of Biology, University of Massachusetts Dartmouth, 285 Old Westport Road, North Dartmouth, MA, USA

## Abstract

**Background:**

Evaluating the limits of adaptation to temperature is important given the IPCC-predicted rise in global temperatures. The rate and scope of evolutionary adaptation can be limited by low genetic diversity, gene flow, and costs associated with adaptive change. Freshwater organisms are physically confined to lakes and rivers, and must therefore deal directly with climate variation and change. In this study, we take advantage of a system characterised by low genetic variation, small population size, gene flow and between-trait trade-offs to study how such conditions affect the ability of a freshwater fish to adapt to climate change. We test for genetically-based differences in developmental traits indicating local adaptation, by conducting a common-garden experiment using embryos and larvae from replicate pairs of sympatric grayling demes that spawn and develop in natural cold and warm water, respectively. These demes have common ancestors from a colonization event 22 generations ago. Consequently, we explore if diversification may occur under severely constraining conditions.

**Results:**

We found evidence for divergence in ontogenetic rates. The divergence pattern followed adaptation predictions as cold-deme individuals displayed higher growth rates and yolk conversion efficiency than warm-deme individuals at the same temperature. The cold-deme embryos had a higher rate of muscle mass development. Most of the growth- and development differences occurred prior to hatch. The divergence was probably not caused by genetic drift as there was a strong degree of parallelism in the divergence pattern and because phenotypic differentiation (Q_ST_) was larger than estimated genetic drift levels (microsatellite F_ST_) between demes from different temperature groups. We also document that these particular grayling populations cannot develop successfully at temperatures above 12°C, whereas other European populations can, and that increasing the muscle mass development rate comes at the cost of some skeletal trait development rates.

**Conclusions:**

This study shows that genetically based phenotypic divergence can prevail even under conditions of low genetic variation and ongoing gene flow. Furthermore, population-specific maximum development temperatures along with musculoskeletal developmental trade-offs may constrain adaptation.

## Background

Evolutionary change in a population can be very fast, with measurable genetic change occurring over only a few generations [1]. However, the ability of animals to evolve to keep pace with rapid climate change remains enigmatic [3,4]. There are a number of factors that might constrain or impede such rapid adaptation. Firstly, low genetic diversity, where small or homogeneous populations lack the genetic "raw material" for adaptation is commonly assumed to constrain adaptation [5]. Secondly, ongoing gene flow is assumed to strongly constrain adaptation, where local adaptations, especially those arising in "sink" demes, are swamped by alleles from interbreeding (source) demes adapted to other environments [6,7]. Various quantitative genetic models have attempted to predict the constraints caused by gene flow under migration - selection balance [8,9] but clearly more empirical tests are needed. Thirdly, genetic drift may strongly constrain adaptive evolution, where small populations suffer from maladaptive genetic differentiation due to influence from random processes [10]. Lastly, there may be costs to adaptation, where agonistic pleiotropy [7] or developmental integration [11] limits adaptation of any given trait because of its interaction with other traits during development.

Demonstrating that natural populations have adapted to climate change or novel habitats is not an easy task [12]. This will require data that either shows, or from which it can be inferred, that traits under study have undergone selection. Also, this selection process should be linked, either directly or indirectly, via known environment and organism interaction mechanisms, to climate change or novel habitat characteristics. Finally, the change in trait values has to be demonstrated to have a genetic basis that is adaptive. A recent review concluded that even though the evidence for climate- and habitat change-driven phenotypic responses in wild populations is indisputable, it is less clear what underlying mechanisms are causing these changes [12]. Indeed, many responses perceived as adaptations to changing environmental conditions could be environmental-induced plasticity. Also, plasticity itself may evolve. Lande (2009) [13] suggested that adaptation actually may occur in two phases, the first being rapid evolution of increased plasticity in the new environment allowing the mean phenotype to quickly approach the new optimum. This first phase is then followed by a slower genetic assimilation.

**Figure 1:**
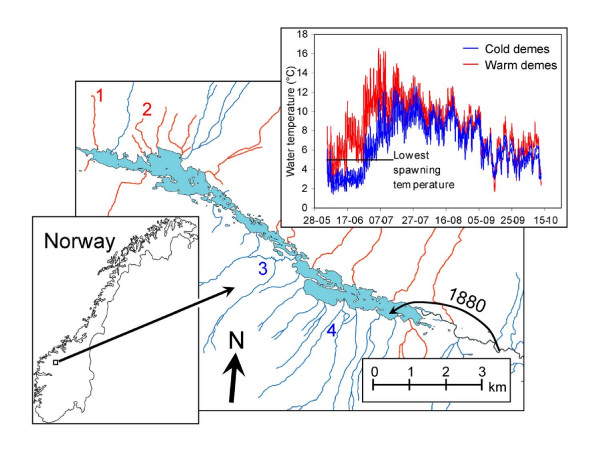
**Study system and stream temperature profiles**. Map showing the location of the study site as well as the 2005 temperature profiles for the four demes involved in the common-garden experiment. Numbers attached to tributaries display the location of the demes (warm demes red numbers, cold demes blue numbers). The 1880 labelling refers to the year of colonization of the lake.

In this study, we examined the ability of a freshwater fish to adapt to conditions relevant to climate change under circumstances that, theoretically, should limit adaptation. In the early 1880s, a man-made connection to the upper reaches of the river Gudbrandsdalslågen, Norway, allowed grayling (*Thymallus thymallus*) to colonize the mountain lake Lesjaskogsvatnet [14] (Figure 1). Later dam constructions severely reduced this opportunity for further invasions of grayling, but grayling from Lesjaskogsvatnet may disperse back to the river system. The exact number of founders is unknown, but it is likely to have been a limited number due to the poor grayling habitats in the upper reaches of the river. Furthermore, analyses of microsatellite DNA have revealed that the source population possesses very low levels of neutral genetic variation [15]. When grayling colonize new lake/river systems, their homing behaviour allows rapid structuring into reproductively more or less isolated demes [16]. The Lesjaskogsvatnet population is currently divided into ca. 20 demes spawning in different tributaries [17]. Microsatellite data from Lesjaskogsvatnet shows significant differentiation among many of the grayling demes in the lake with a significant isolation by distance population structure [18], but the population structure is unstable with the underlying isolation by distance structure in some years being swamped by gene flow [19]. All demes spawning in the tributaries also show clear signatures of recent bottlenecks, but based on the available evidence it is not clear if this is a signal from the original invasion of the lake or more recent independent events [18,19].

Lesjaskogsvatnet is an elongated lake and consists of a shady and a sunny side because of high surrounding mountains. Demes spawning in sunny-side tributaries spawn up to four weeks earlier and their eggs and larvae have a considerably higher development temperature as water temperatures rise much faster than in shady side tributaries (Table 1 and 2), thus providing natural replicates of "warm" (sunny) and "cold" (shady) tributaries (Figure 1, Additional file 1). The young grayling stay in the tributaries for 2-3 months (allopatric phase), after which they mix with other demes within the lake (sympatric phase). Once in the lake, the juveniles from both warm and cold demes will compete over the same food resources striving to access enough food to build up sufficient resources for surviving their first 6-7 month winter, when feeding opportunities are strongly reduced. For juvenile freshwater fish in temperate areas, large body size, and hence larger energy reserves has repeatedly been shown to increase accession of food and winter survival [20]. Being born weeks later than warm-deme embryos, cold-deme embryos face a tremendous time-constraint as they have shorter time available for attaining a certain size and/or energy level to make it through the first winter. On top of that, the cold-deme embryos need to grow and develop faster under colder conditions (Table 2).

**Table 1:** Environmental characteristics for the ancestral (Gudbrandsdalslågen) and colonized systems (warm streams in bold and cold in *italics*)

Location (Figure 1)	Mean width (m)	**Mean discharge (m**^**3**^**/sec)**	**Mean water temperature during spawning period (°C)**^**1**^
Gudbrandsdalslågen	14.3	10.1 ± 15.3	6.3 ± 1.4 (1992-2001)
**Bella (1)**	**1.3**	**0.09**	**6.1 ± 0.7 (2005-2006)**
**Søre Skotte (2)**	**0.7**	**0.02**	**5.8 ± 1.7 (2003-2007)**
*Hyrjon (3)*	*5.0*	*0.25*	*3.2 ± 1.1 (1996-2007)*
*Valåe (4)*	*5.8*	*0.42*	*2.9 ± 0.9 (1996-2007)*

^1^01 June-10 July (years covered are provided in parenthesis)

**Table 2:** Estimated development temperature sums (*D*). *t*_min _refers to the date at which the stream water temperature exceeds 4°C for the first time during a given year.

	*D *(mean ± s.e.) °C			
			
Development period	Warm demes	Cold demes	*F*^1^	*P*
*t*_min _+ 2 weeks	113.3 ± 2.7	91.1 ± 3.1	22.10	<0.0001
*t*_min _+ 4 weeks	242.2 ± 4.2	220.0 ± 4.5	10.64	0.0033
*t*_min _+ 6 weeks	394.0 ± 5.1	371.1 ± 5.4	5.04	0.0342

^1 ^df = 1,24 for all tests. One-way anova.

To test for genetic differentiation among demes in developmental traits, we conducted a common-garden experiment using embryos and larvae from two cold and two warm demes (Figure 1) exposed to 8°C and 12°C water temperature (see Methods section). This setup allows for testing for genetic differences among demes exposed to the two temperatures, but does not explicitly test for local adaptation. We chose 8°C as the common-garden temperature since embryos from all demes would experience this temperature in nature soon after fertilization. The 12°C treatment level was chosen to test the effect of a high incubation temperature within a potential range that may result from global warming. Since growth rate in fishes, as measured in length or mass, represents primarily growth in the musculoskeletal system [21], and previous studies have reported physiological or performance trade-offs in growth-rate evolved populations [22-24], we investigated differences in musculoskeletal developmental rate evolution in more detail. We analyzed both muscle-fibre development and skeletal ossification in the four demes. The common-garden study design employed in this study allows for assessment of parallelism in divergence for pairs of demes belonging to the same temperature regime. In order to evaluate the relative role of natural selection and genetic drift to observed phenotypic differentiation, we used information about microsatellite-based F_ST _as a measure of drift-generated genetic differentiation. These estimates were compared with the analogous trait-wise Q_ST_-estimates measuring genetic differentiation in phenotypic traits, where Q_ST _>F_ST _suggest differentiation due to divergent selection and Q_ST _<F_ST _suggest stabilizing selection [25]. By combining information on 1) degree of parallelism in trait expression between demes from same temperature group with 2) the degree of congruence between the observed and expected direction of trait differentiation and 3) pair wise Q_ST _vs F_ST _comparisons we are able to draw inferences about signatures of local adaptation.

## Results

At 12°C, no embryos survived past 5 days post hatch, while at 8°C many embryos survived to 30 days post hatch (when the experiment was terminated).

### Individual growth processes

**Figure 2:**
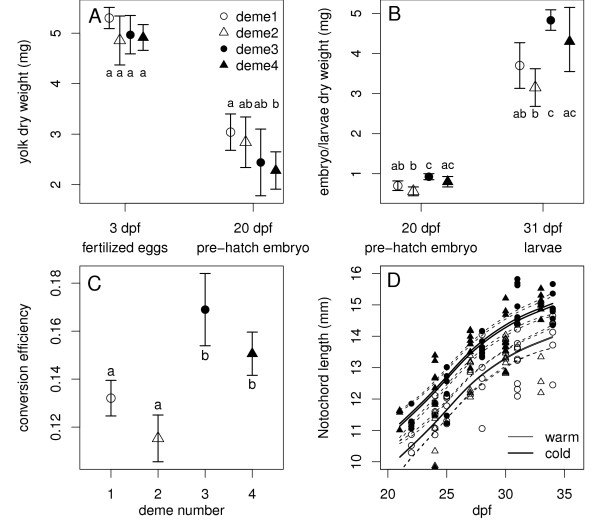
**Growth trajectories and yolk-to-body mass conversion efficiencies**. Mean (± 95% confidence intervals) dry weights of total embryo, yolk sac and hatchlings at 3, 20 and 31 days post fertilisation (*DPF*) **A**. rate of yolk absorption, **B**. rate of embryo and hatchling growth. Similar letters indicate demes that have non-significant differences in conversion efficiencies (Tukey-Kramer HSD pairwise contrast tests). **C**. Mean (± 95% confidence intervals) yolk-to-body mass conversion efficiency of grayling embryos from warm and cold demes raised under similar conditions (8°C). The conversion efficiency was estimated as the ratio of embryo dry weight at 20 *DPF *on egg dry weight at 3 *DPF*. Similar letters indicate demes that have non-significant differences in conversion efficiencies (pair-wise t-tests). **D**. Notochord length-at-age for warm and cold deme post-hatch grayling larvae raised under similar conditions (8°C). Lines correspond to the GAM-predicted growth trajectories for each deme (see Additional file 2). Dashed lines correspond to 95% confidence bounds. The GAM explained 77% of the variation (group effect: *F*_1,144 _= 803, *P *<< 0.0001).

While egg diameter and dry weights were not different among demes (*F*_3,24 _= 1.93, *P *= 0.15), a comparison of mean dry weights of fertilized eggs, pre-hatch yolk and embryos (weighed separately), and pre-feeding larvae revealed that the cold deme embryos grew faster and absorbed yolk faster than warm deme embryos (Figure 2A, B). Yolk conversion efficiencies (i.e., how efficiently yolk mass was transformed to body mass) were significantly and consistently greater in cold demes than in warm demes (Figure 2C). Notochord length (*NL*) measurements from digital images of all daily samples from each deme (n = 6-10/deme/day) confirmed that size-at-age differences were found throughout larval development, replicated in both cold demes and warm demes (Figure 2D). A generalized additive model fitting size-at-age as a function of days post fertilization (*DPF*) and temperature group, revealed that all demes had parallel growth trajectories (i.e., little support of a DPF*deme model as AIC was 67 levels larger than the *DPF+deme *model) where cold-deme embryos started out as 1.00 mm longer (s.e.m = 0.11, *t *= -8.99, *P *< 0.0001, Additional file 2) at 21 *DPF *and ended up as 1.00 mm longer after 13 days.

### Developmental trait trade-offs

**Figure 3:**
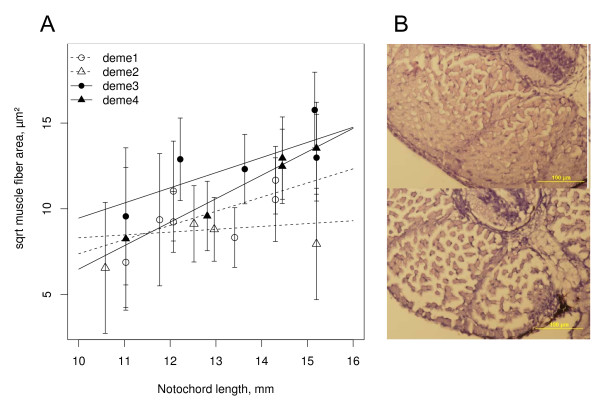
**Differential muscle development in relation to embryo size**. **A **Average (± SD) square root muscle fiber areas (micrometers^2^) at length for cold vs warm deme post hatch larvae. Lines correspond to deme-wise predictions retrieved from the most supported GLM (see Additional file 2). **B**. Histological transverse sections from a 13.6 mm cold-deme larva (upper photo) and a 13.4 mm warm-deme larva (lower photo) illustrating the larger, more numerous fibers observed in cold-deme larvae.

**Figure 4:**
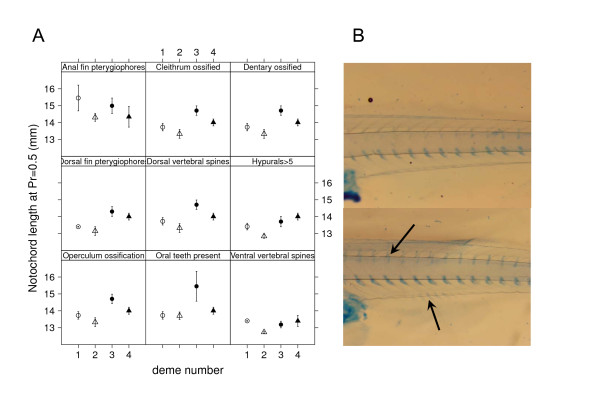
**Differential skeletal development in relation to embryo size**. **A **Deme-wise GLM-predicted notochord lengths (± 95% confidence bounds) at 50% probability for reaching certain stages in nine skeletal development traits (see Additional file 2). Cold deme larvae are larger in most of the skeletal traits. Skeletal traits are: Anal-fin pterygiophores initiated; Cleithrum ossified; Dentary ossified; Dorsal vertebral spines initiated; Dorsal-fin pterygiophores initiated; Hypurals>5; Operculum ossification initiated; Oral teeth present; Ventral vertebral spines initiated. **B**. Cleared-and-stained caudal areas from 14.9 mm cold-deme larva (upper photo) and 15.0 mm warm-deme larva (lower photo) illustrating the relatively delayed skeletal development of the cold deme larvae. Lower arrow points to forming anal spines and upper arrow points to anterior dorsal vertebral spines, neither of which are present in the cold-deme larvae.

In histological cross-sections of muscle made at a homologous point (the anus), we found that faster-growing cold deme individuals had a significantly larger average muscle fibre area at a given age and size (Least Square Mean (LSM) ± 2s.e.m. from generalized linear model (GLM): *NL *= 14 mm: LSM_cold _= 142.6 ± 12.4 μm^2^, LSM_warm _= 115.4 ± 10.3 μm^2^) (Figure 3). In contrast, rate of skeletal developmental was delayed in cold deme fish when compared with similar-sized fish from warm demes. Both axial and cranial skeletal features developed faster in cold demes (all 9 traits had lower group effect coefficients for warm demes, GLM-derived *P*_temperature group_-values ranging from < 0.0001 to 0.061). Hence, cold deme individuals reached a certain trait expression probability earlier than warm-deme individuals. However, in 7 out of 9 traits (GLM-derived *P*_temperature group_-values ranging from 0.003 to 0.021, Additional file 2), the skeletal development did not "keep up" proportionately with notochord length in the cold demes (Figure 4).

### Qst vs Fst

Pair-wise differences in analogous F_ST _and Q_ST _values are displayed, for three traits, in Additional file 3. In total, 88% (28/32) of the warm vs cold deme pair wise comparisons are consistent with divergent natural selection and 12% could not be distinguished from the pattern expected under genetic drift. For comparisons between demes within the same temperature group only 19% (3/16) of the comparisons indicated divergent selection, whereas 81% could be attributed to convergent evolution or drift. Because 5 out of 6 F_ST _values had confidence bounds that overlapped with zero, inferences on eventual convergent evolution (i.e., F_ST _>Q_ST_) could not be made.

## Discussion

This study provides compelling evidence that individual growth- and developmental rate and efficiency can evolve over fewer than 22 generations under conditions widely theorized to impede local adaptation. In particular, the degree of parallelism in the direction of divergence for most of the traits investigated reveals compelling signals of adaptation-driven differentiation. Cold deme individuals were shown (with rare exception) to consistently have a more efficient yolk-to-body-mass conversion efficiency, a higher somatic growth rate and a higher skeletal- and muscle mass developmental rate compared to warm-deme individuals. All of this is in accordance with predictions on expected direction of divergence caused by temperature differences between deme habitats. Since some divergence has take place and also since this divergence is according to the predicted direction of trait evolution, we conclude they are not severely constrained or limited in their adaptation to temperature differences - even under conditions with substantial gene flow. However, we cannot rule out that they may have been constrained below what would be the optimum divergence. One should bear in mind the spatial scale for this particular study system - the demes are separated by just 2-6 km during the reproductive period. As a consequence, any spawning site can easily be accessed by any adult grayling individual in the lake during just one day (e.g., a 30 cm individual can at a cruising speed of 0.25 body lengths per second reach more than 6 km during a day). Still, we know from previous studies that local adaptation can occur within such spatio-temporal scales [26-28]. In fact, our findings of temperature-related divergence of developmental and ontogenetic timing traits are very much in agreement with findings in other salmonid systems with recent origin. Very similar to our findings, in a system very comparable to our grayling system, Hendry et al. [29] found that development rate was fastest for progeny of late spawning sockeye salmon (*Oncorhynchus nerka*) demes in Lake Washington. Further, yolk conversion efficiency was related to natural incubation temperatures. This adaptive divergence pattern had arisen in only 9-14 generations, within the same lake system with demes separated by less than 10 km. Similarly, Kinnison et al [31] found that early-life growth rates differed between recently (90 years) established populations of chinook salmon (*Oncorhynchus tshawytscha*) in New Zealand. However, for the same populations embryo development rates did not differ [30]. As a consequence, due to temperature differences, the emergence dates could differ by up to six weeks between rivers. Hence, salmonids seem to have the potential to adapt to novel temperature regimes over fewer than 10 generations, even at spatial separation scales smaller than 10 km. But it is not always the same traits that diverge, and therefore one cannot claim that adaptation is inevitable.

The patterns of differentiation in larval developmental rate and efficiency in relation to temperature in the Lesjaskogsvatnet grayling supports findings in a number of other studies; this include 1) the relationship between temperature environment and individual growth rate [32] and development rate [33,34], and 2) trade-offs between muscle growth and skeletal differentiation [22,35-37]. Taken together these results suggest that differentiation of individual growth, metabolism and development according to local environmental temperature is likely due to local adaptation. However, we also show that there are trade-offs among several of the musculoskeletal traits studied here. Rapid development of muscle comes at the cost of decreased development of functional structures. Thus, the adaptation of the various traits to different environmental conditions seems constrained by phenotypic (and probably genetic) correlations. Such intrinsic constraints, which probably are very common in biological systems, will, potentially, lead to a slower evolutionary response to environmental change and it will make predictions about the direction and speed of change difficult.

Temperatures in Nordic countries are expected to rise at higher-than-average rates over the coming decades [38], requiring Nordic lake fish populations, with no opportunity to migrate, to adapt (e.g., [33]). Recent studies from marine and terrestrial systems predict that the adaptive response to increase in temperature will be, firstly, that individuals will maximize growth and energy efficiency at the expense of ranges of thermal tolerance [39]. These studies also suggest that species at higher latitudes have broader thermal tolerance and are living in climates that are currently cooler than their physiological optima, and, as such, warming may even enhance their fitness [40]. For our specific grayling population under study, we found that the embryos were unable to successfully develop at temperatures above 12°C - indicating a narrow thermal tolerance. Grayling populations elsewhere in Europe clearly are able to develop successfully at these temperatures [41]. Field observations (unpublished information) of high clutch mortality following spring temperature spikes support the laboratory findings of mortality at high temperatures. In addition, both common-garden rearing experiments and long-term field observations of spawning temperatures indicate that grayling embryos cannot survive below 5°C, further illustrating the narrow range of temperatures in which embryos of this grayling population can survive. Hence, will the grayling in Lesjaskogsvatnet be able to adapt to increasing temperatures? The apparent stenothermy suggests that phenotypic plasticity alone may not be sufficient for the grayling to cope, especially because we found no inter- or intra deme variation to successful development at 12°C. Rather, the populations would need to evolve either a change in temperature responses or a change in spawning time (see Wedekind and Küng [42] for a grayling example). Indeed, a change in phenology is probably the most rapid way for a population to adapt to new climatic conditions [4,43]. There are very few reported examples that have demonstrated a genetic shift in thermal optima or thermal tolerance [43]. However, since the change in temperature will occur over decades, a gradual adaptation to the gradually changing seasonal water temperatures is likely through both change in phenology (spawning time) and change in temperature tolerance range. This mixture of changed phenology and gradual adaptation to slightly higher water temperatures is a likely evolutionary scenario since the water temperatures experienced by the embryos would be only slightly altered if the grayling start spawning earlier in spring. The prospects for an earlier spawning time is good as the current spawning time is very late (June) compared to other European populations (March-May [45]).

Previous studies of salmonids have shown that low temperatures may delay and prolong myogenic regulatory factor expression and muscle differentiation in the embryos [46]. Our result showing accelerated muscle, but delayed skeletal differentiation in low-temperature demes is indicative of a heterochronic evolutionary trade-off where resources are allocated competitively between growth and developmental processes in the musculoskeletal system. These types of internal trade-offs have been hypothesized previously to affect evolutionary divergence patterns based on macroevolutionary patterns and cellular level mechanisms [22,35-37]. Since muscle and skeletal system development is linked to swimming performance in fishes, our data also suggest a connection to the emerging theory regarding performance trade-offs with fast intrinsic growth rates [22,23].

Previous reports show that isolated grayling populations derived from a single ancestral population rapidly adapt growth rates to local environmental conditions, even under strong influence of genetic drift [14,47,48]. We report here that for sympatric grayling demes in Lesjaskogsvatnet only a few weeks of temperature-specific isolation per year seems to have been sufficient to cause genetic differentiation. Because we are working with evolution in wild populations, we cannot clearly distinguish whether temperature is directly and solely driving the divergence or whether there are indirect temperature effects (e.g., by interactions with the length of the growing season). However, other salmonid studies demonstrate direct temperature effects during embryogenesis that cause growth and muscle phenotype differences that persist into adulthood - even if the later ontogeny is expressed under similar environmental conditions [49]. In a compelling study on two Atlantic salmon populations in Scotland, Johnston and co-workers [50] demonstrated a higher yolk-to-body mass conversion efficiency and larger muscle fiber recruitment during embryogenesis in a cold-adapted population that was compared to a warm-adapted one. The cold-adapted embryos did particularly well under thermal conditions similar to the one in their native stream - indicating that the differences observed could be attributed to local adaptation. Our experimental design only allows for drawing inferences about genetic differentiation - and does not test for local adaptations directly. However, because of the replicated divergence pattern among demes observed in this study (parallelism), and also the direction of the divergence (as predicted from previous studies), we argue that the differences observed are largely due to local adaptations. The observed divergence is not likely to be primarily due to genetic drift because microsatellite-based F_ST_-values were generally found to be smaller than Q_ST _values between deme pairs of differential temperature origin, and not different between deme pairs of similar temperature origin (Additional file 3). However, as pointed out in the methods section, one should interpret both the estimated F_ST _and Q_ST _values with caution as there are many sources of bias that may substantially have affected them both [51,52].

The ability to successfully adapt to new environments is assumed to be facilitated by large genetic variation [10]. Hence, bottleneck situations may therefore potentially affect a population's prospects for future adaptation. However, studies suggest that bottlenecked populations with low genetic diversity have a greater chance of avoiding extinction if they have opportunity for population expansion [53]. Annual gillnet catches of grayling in Lesjaskogsvatnet include up to 40,000 individuals, thus a post-colonization population expansion has occurred in this system. This opportunity for population expansion, following from the fact that the grayling colonized a system with available niches, may have played a major role in the observed divergence process and avoidance of local extinction. Even though gene flow most often is considered to hinder local adaptation [7] this is not necessarily always the case [54]. For instance, Garant et al. [55] predict that an intermediate level of gene flow will allow the greatest adaptive divergence. Gene flow among inbred demes may increase the evolvability of these demes, and as such enhance the short-term adaptation [10]. Detailed studies on not only among-deme dispersal patterns, but also offspring viability of between-deme crosses, would be needed to explore the consequences of gene flow for genetic variation and responses to selection.

## Conclusions

This study provides evidence that natural selection is sufficiently powerful for temperate lake fish populations to adapt to novel temperature regimes within 22 generations, even under conditions with low genetic variation and under influence of gene flow. We also provide evidence pertinent to the IPCC-predicted global warming; namely that current genetic variation and constitution related to embryo development may not be sufficient to allow for adaptation to such a high temperature increase. However, by altering the spawning time the temperature during early development may change sufficiently little for adaptation to occur. These types of integrative studies, where genetic differentiation, ecological opportunities, and physiological limits are explicitly considered, will be essential in evaluating the capacity for populations to adapt to global warming.

## Methods

### Common-garden experiment

Parents (7-15 males and 7-15 females from each stream) were sampled during the spawning run by using fyke nets in the streams. The warm streams were sampled during 2 to 6 June. The grayling in did not start spawning until 12 June in the cold streams and samples from these ones were retrieved during 12 to 14 June. They were assumed to represent random samples from their respective deme. Eggs and sperm were stripped from these ripe fishes and artificially fertilized *in situ*. Equal amounts of eggs (0.2 L) from each female were mixed together at deme level and this mixture was fertilized using a sperm mixture made up from equal amount from each male from the same deme. Fertilized eggs were kept at deme level in separate perforated plastic jars in 40 L containers and brought to the University of Oslo by car (total transportation time 6 h). Here the fertilized eggs were held in small floating cages within 1 × 1 m tanks and reared at two temperatures (8 and 12°C) in the laboratory for 33 days, until yolk absorption (*sensu *[47]). Each deme was triplicated at each temperature. Dead eggs and larvae were registered and removed from the cages every day.

### Trait measurements

Daily samples of ten individuals were taken haphazardly from the rearing units containing individual clutches and were preserved in 10% buffered formalin. Seventy individuals were selected for comparison of dry weights (5-7 individuals × 4 demes × 3 selected days). Samples were dried for 7 days at 60°C. Weights were taken from 3 days post fertilisation (*DPF*, early egg), 20 *DPF *(prehatch; yolk and embryo were separated, dried, and weighed separately), and 31 *DPF *(larvae). Two of the largest specimens from each of the daily samples were taken for histological analysis (*n *= 80; 20 per deme). Largest specimens were selected because we reasoned that they were most representative of the healthy growth potential of the group. Average sizes were considered less informative as they are skewed by sickly or developmentally delayed individuals. All specimens were measured for notochord length (*NL*), and then one size series was used for transverse sectioning and another size series for wholemount clearing and staining.

### Growth and developmental trait comparisons

NIH Image software http://rsb.info.nih.gov/nih-image/ was used to obtain notochord length data from digital images taken of each sample (*n *= 5-10/day/deme/temperature).

For sections, samples were dehydrated through an alcohol series, then soaked in xylene (12 min × 3) and embedded with paraffin wax at 62°C (45 min × 3). Transverse sections of the body were taken from each sample at the anus (8 μm). Sections were dried overnight, then rehydrated, stained with Haemotoxylin and Eosin-Y, dehydrated through an alcohol and xylene series, and mounted in Depex medium. Digital images of sections were captured and measurements were taken using NIH Image software. From muscle cross-sections, selected well-defined fiber areas (*n *= 10-112 per individual) from a dorsolateral myotomal segment were measured by drawing an outline around individual fibers. Fibers from the central region of this myotomal segment were haphazardly selected. Wholemount clearing and staining allowed visualization of skeletal development. Formalin-fixed specimens were dehydrated through an alcohol series over one day, then trypsin digested and stained in Alcian Blue (cartilage) and Alizarin Red (bone) following standard protocols [56]. Each sample was examined under dissecting microscope for appearance of individual skeletal elements (cleithrum, dentary, gill arches, operculum, oral teeth), and meristic elements (number of dorsal or ventral vertebral arches, number of dorsal or anal fin pterygiophores, number of hypural elements in the caudal fin skeleton) were counted as they formed (Figure 4).

### Estimation of deme and group effects

The 12°C treatments showed high mortality and high rate of malformations. There was a much higher percentage of gross developmental disorders at 12°C than at 8°C (61% vs 24%), with no obvious difference between cold and warm demes. As a consequence, statistical analyses were performed using data from the 8°C treatment only. The statistical analyses were performed using various generalized linear models (GLM) with link functions depending on the nature of the response variables (*i.e.*, continuous or binomial). In general, all traits were analysed using notochord length and/or days post fertilization as predictor variables - in addition to either deme or temperature-group effects. The rationale for doing so is because the traits analysed change in value expression both as function of size relationships (*NL) *and of development time (*DPF*). These variables were therefore included as covariates so as to study whether demes and/or temperature groups differentiated in covariate-adjusted trait values - indicating genetic differentiation under the prevailing common-garden conditions. The covariate effects were modelled as fixed effects as eventual deme*covariate interaction effects were of particular interest, potentially indicating genetic differentiation in allometric and/or development trajectory relationships. In order to explicitly explore parallelism in trait responses for demes belonging to the same temperature group, deme effects were nested under temperature group effects. Hence, the core model for all analyses was:

(1) $$Y_{\text{ijk}}=\alpha_{\text{i}}+\alpha_{\text{ij}}+\beta_{\text{1ij}}\;NL_{\text{k}}+\beta_{\text{2ij}}\;DPF_{\text{k}}+\beta_{\text{3ij}}\;DPF_{\text{k}}\;NL_{\text{k}}+\varepsilon_{\text{ijk}}$$

where *Y*_ij _is the trait value for individual k in deme j (j∈{1,2,3,4}) and group i (i∈{warm, cold}). α_ij _is the group effect for deme i, β_1ij _and β_2ij _are slope estimates for the effect of notochord length and time since fertilization, respectively. β_3ij _represents the interaction effect between the two covariates. In general, the residual variance was assumed to be distributed *N*~(0,1) under the applied link function. For binomial traits the logit link function was applied. The most supported model structure was selected using Akaike Information Criterion [57]. All GLM analyses were performed using the glm procedure in R (version 2.10.1, [58]).

For extracting estimated notochord lengths (and corresponding standard errors) at a certain trait expression probability (i.e., Pr = 0.5) in some binomial skeletal traits the 'dose.p' procedure implemented in the R library MASS was applied to the selected trait-specific GLM models [59].

For the trait 'muscle fiber area' many fiber areas were measured per individual (*n *= 29 ± 32, mean ± s.d.). Hence, when modelling the effect of *NL*, group and deme for this trait the within-individual variance component had to be taken into account. This was done by adding ID as a random effect using a linear mixed model approach [60]. Due to the imbalanced data, the candidate models were fitted using the REML method implemented in the lme4 library in R [58]. The most supported model structure was chosen based on AIC.

In order to test for differentiation in notochord growth trajectories, a generalized additive model (GAM) [61] was used as no *a priori *parametric model of growth trajectories were available under the prevailing experimental conditions. This GAM approach enabled selection of the most parsimonious non-linear growth model by choosing the model involving the lowest degrees of freedom of the smoothing functions. The model selection was performed using generalized cross validation criterion [62] as implemented in the MGCV library in R (version 2.10.1 [58]).

A test of total dry weight of yolk and embryo as function of days since fertilisation and deme was conducted using ordinary two-way ANOVA with days since fertilisation coded as an ordinal variable with levels 3, 20 and 31 days since fertilization.

### Q_ST _and F_ST_

In order to assess the relative influence of genetic drift and natural selection to observed phenotypic differences between pairs of demes, we used a method recently suggested by Sæter et al. [63]. Using this approach, the range of potential Q_ST_-estimates can be assessed even under conditions where the additive genetic components are not explicitly available. The authors argue that their method allows for Q_ST_-estimation (or rather P_ST _estimation) even under ordinary field conditions where no information about family structure is available. Our experiment was conducted under common-garden conditions, but, owing to the lack of a family design, no estimates on additive genetic variance components could be derived. However, the between-population variance, or between-deme in our context, could be estimated. Following this approach, Q_ST _was estimated as:

(2) $${\text{Q}}_{\text{ST}}=\frac{g\mathrm{var}(\text{pop})}{g\mathrm{var}(\text{pop})h^{2}\mathrm{var}(\text{error})}$$

where g is the assumed additive genetic proportion of differences between populations, h^2 ^(narrow-sense heritability) is the assumed additive genetic proportion of differences between individuals within populations, var(pop) is the observed between-population variance component and var(error) is the observed within-population variance component. The ranges of likely Q_ST_-estimates were simulated across different values of g and h^2^, with g ranging from 0.05 to 1 and h^2 ^ranging from 0.05 to 0.8. Owing to difficulties in assessing reliable residual variance components for binary traits (3 of the skeletal traits), we were able to estimate Q_ST _for 8 of the 11 traits involved in this study. The variance components were estimated from mixed models fitted using the lmer-procedure in the lme4 library in R, version 2.10.1 [58]. In all models, fixed effects of *NL *and/or *DPF *were included and demes were coded as random effects.

Estimates of inter-population neutral genetic differentiation (F_ST_) and their 95% confidence intervals were obtained using the variance component approach (providing the F_ST _estimator theta) and bootstrapping of loci with program FSTAT version 2.9.3.2. http://www2.unil.ch/popgen/softwares/fstat.htm. In total, 18 polymorphic loci were used for this estimation (Additional file 4). The DNA used in the analysis was sampled from fin clips of 14-43 individuals from each deme during spawning time in 2004.

Inferences about whether a trait had diverged mainly due to natural selection were drawn, as suggested by Whitlock 2008 [52], based on whether the simulated Q_ST_-values overlapped with the overall F_ST _probability distribution (i.e., 95% confidence bound across all 18 loci). However, this Q_ST_-F_ST _comparison is not to be considered a precise test for natural selection, but rather as a coarse assessment to explore if some traits and deme comparisons show consistent patterns of differences in the two metrics. Both the F_ST_-values and Q_ST_-values (in particular) estimated for this study are likely to be imprecise. The number of demes and number of loci involved were too low to produce reliable estimates (should have been >10 and >20, respectively [52]). The lack of a breeding design and possible common-garden experimental environment artefacts are sources of potential bias for the Q_ST _estimates.

## Authors' contributions

KDK, TOH, and LAV conceived the study. KDK, TOH, JJ, and FG contributed to the collection and analysis of the data. All co-authors contributed to refining the analyses, interpreting the results, and improving the manuscript. All co-authors read and approved the final manuscript.

## Supplementary Material

Additional file 1 **Analysis of environmental differences between streams with particular attention to water temperature and consequences for timing of spawning and development temperatures**. A detailed description of a dynamic factor analysis (DFA) that utilize time-series data on water temperatures covering four years of data. The analysis groups the streams into cold and warm ones. This file includes **Figure A1 **where factor analysis part of the DFA is plotted for the different streams involved in the study. This file also includes estimates of spawning-time segregation using a generalized additive modelling approach where time and temperature sums are included as predictor variables. Includes **Figure A2 **showing the GAM model predictions for the timing of spawning. Click here for file

Additional file 2 **Parameter estimates and model fit statistics for the most supported models fitted to estimate group effects for 11 traits involved in the study (Table A1)**. Click here for file

Additional file 3 **Qst vs Fst comparisons between pairs of the four demes included in the study (Figure A3)**. The plots comprise sensitivity plots of Q_ST_-values under various g and h^2 ^settings. Left panels show simulations for muscle fiber area, middle panels for notochord length and right panels for the skeletal trait hypurals (as an example of skeletal trait). Horizontal dotted lines indicate the confidence interval for analogous F_ST_-estimates and numbers provided in the figures correspond to mean F_ST _values and expected Q_ST _values, respectively. The expected Q_ST _corresponds to estimates where g = 0.8 and h^2 ^= 0.3. Click here for file

Additional file 4 **Characteristics of microsatellite loci applied in the study. (Table A2)**. Click here for file
