# Supplementary material for: Contemporary temperature-driven divergence in a Nordic freshwater fish under conditions commonly thought to hinder adaptation
Source: BMC Evol Biol. 2010 Nov 11;10:350. doi: 10.1186/1471-2148-10-350 (PMC2994878; doi:10.1186/1471-2148-10-350)
Supplement: Additional file 1 — Analysis of environmental differences between streams with particular attention to water temperature and consequences for timing of spawning and development temperatures. A detailed description of a dynamic factor analysis (DFA) that utilize time-series data on water temperatures covering four years of data. The analysis groups the streams into cold and warm ones. This file includes Figure A1 where factor analysis part of the DFA is plotted for the different streams involved in the study. This file also includes estimates of spawning-time segregation using a generalized additive modelling approach where time and temperature sums are included as predictor variables. Includes Figure A2 showing the GAM model predictions for the timing of spawning. [file 1471-2148-10-350-S1.PDF]

Additional file 1:

Differences between ancestor and colonized environments

The Gudbrandsdalslågen grayling individuals that colonized Lesjaskogsvatnet during 1880s (Figure 1) entered an environment very different from the one from which they originated. Firstly, they came from an entirely riverine (lotic) environment and entered a lake (lentic) environment. Second, even if embryos and early larvae live in a lotic environment in both the ancestral and colonized system, these lotic environments are very different both with respect to temperature and discharge (Table 1). The warm streams seem to have a temperature regime more similar to the ancestral river than the cold streams, but they are clearly more different with respect to size and discharge than the cold streams. As a consequence of these differential environments between ancestral and colonized habitats, pre-adaptation of the founders is highly unlikely.

Water temperature analysis

Assignment of streams into temperature groups (warm or cold) was based on June-August water temperature sums and stream size in a *k*-mean cluster analysis [1]. In order to explore the temporal consistency of these assignments, we commenced a dynamic factor analysis (DFA)[2] on the 45 time series of temperature data available for the 2003–2007 period. DFA is a multivariate time-series analysis technique used to estimate underlying common patterns in a set of time series. We use this technique in order to 1) assess the number of common temporal trends (M) for the four years with detailed temperature data (retrieved from HOBO® temperature loggers) and 2) exploring the factor loading (z) pattern we can infer the relevance of the predefined temperature group assignments of the streams involved. The mathematical formulation used in DFA can be written as:

$$T_{jt} = z_{j1}\alpha_{1t} + z_{j2}\alpha_{2t} + \ldots + z_{jM}\alpha_{Mt} + e_{jt} \qquad (1)$$

where T_{jt} is the mean water temperature for stream j at day t, α_{kt} is the kth common trend, z_{jt} is the factor loading and e_{jt} is noise. In matrix notation, this can be written as

$$\mathbf{T}_t = \mathbf{Z}\boldsymbol{\alpha}_t + \mathbf{e}_t \qquad (2)$$

where $\mathbf{T}_t$ is a $N \times 1$ vector containing the temperature values of the N time series at time t, $\boldsymbol{\alpha}_t$ represent the values of the M common trends at time t, and $\mathbf{e}_t$ is a $N \times 1$ noise component, which is assumed to be normally distributed with mean 0 and covariance matrix $\mathbf{R}$. The $N \times M$ matrix $\mathbf{Z}$ contains the factor loadings and determines the exact form of the linear combinations of the common trends. The aim of DFA is to model all the time series using as low M as possible and still explain the data reasonably well. The optimal M is selected using the model selection metric Consistent Akaike's information criterion (CAIC) [2]. After finding the most supported M, we compare the stream group assignments with the factor loadings. All DFAs were performed using the *R*-based Brodgar software version 2.5.4 (http://www.brodgar.com/).

The DFA analysis revealed that 1) in three out of four years the CAIC favoured two common trends for the time series (three trends in 2006), and 2) it clearly demonstrated that the streams have been relevantly assigned to their respective groups. There is one exception though, the stream BRA, that group together with warm streams for two of the four years (Figure A1). For the four streams involved in this study all of them locate consistently within the cluster of their group over all four years (Figure A1). Hence, in general, the grouping of streams into warm and cold streams reflects a real environmental difference, not only when taking the mean water temperature into account [1], but also when analysing the entire temporal temperature profile (the current DFA analysis).

Consequences of temperature differences on incubation conditions

The documented temperature differences will affect timing of spawning and is also likely to affect the incubation temperatures. Both our observations and observations on grayling further down the Lesjaskogsvatnet drainage [3] show that spawning does not occur at temperatures below 5°C and that the warm streams reach 5°C earlier than cold streams in most years (Figure 1).

In order to address the possibility that incubation temperature differs between the two groups of streams, we fitted generalized additive models (GAMs; see section later in this text) to spawning date data. For each stream, we constructed binominal arrays of observations where dates with no observed spawners in the stream where assigned “0” and days with observations of spawners where assigned “1”. This response array was fitted effects of day number (10 June was set to zero, $t_{10\ \mathrm{June}}$=0) and temperature sum (D), where D was defined as

$$D_t = \sum_{t_{\min}}^{t} T_t - 4 \qquad (3)$$

where $t \geq t_{\min}$ and $t_{\min}$ is the day at which the daily mean temperature exceeds 4°C for the first time, and T_t is the mean temperature during day t. We restricted the analysis to the 20 May to 12 July period, which corresponds to 5 days outside the outer range dates for spawning observed over the 1995 to 2007 period. The binominal GAMs were fitted using a logit link function and the predictors were modelled as thin plate multidimensional splines [4] where t and D were allowed to interact with a maximum number of degrees of freedom (k) set to 5 (i.e., in GAM-notation, s(t,D, k=5)). In addition, stream type was included as a fixed nominal effect, with the opportunity for this effect to interact with both t and D. The most supported GAM structure was selected by generalized cross validation [5]. Once the most supported model was established, we used this model for predicting spawning dates (defined as the 50% probability isocline), and from these spawning date predictions we estimated the incubation

temperature sums (*DI*), defined as the temperature sum over the date-at-spawning to 15 September period. We also estimated *DI*s over 2 week, 4 week and 6 week incubation periods. These *DI*s were compared between warm and cold streams so as to evaluate the degree of *DI* differentiation using one-way ANOVA.

The most supported GAM did not include a stream-group effect, indicating that all Lesjaskogsvatnet grayling follow the same rule of action with respect to when to enter the streams for spawning. The model explains more than 85% of the deviance and the estimated combined smoothed effect (estimated degrees of freedom = 2.98, χ^2 = 177.6, P<0.00001) predicts entrance to the streams to require a higher *D* at earlier dates than later dates for a given probability of entrance (Figure A2). For instance, a 90% probability of entrance would require *D*-values of 39°C *vs* 5°C if the fish were to enter the stream on 5 June compared to 25 June, respectively. Evidently, only warm streams warm up sufficiently early to reach such high temperature sums in early June. The mean (±s.e.) spawning date was –1.6±0.9 (June 8) and 5.6±1.1 (June 16) for warm and cold streams, respectively, which is significantly different ($F_{1,33}$=24.82, P<0.0001).

The estimated incubation temperature sums were significantly higher in warm streams than cold streams in all combinations tested (Table 2). However, the main difference occurs during the first 14 days following spawning. Over this period the temperature sum difference is more than 22°C, and this difference remains up to 6 weeks. Because warm deme individuals will have a potentially longer period for early development (assuming they leave the streams by mid September (own observations, T.O.H.), the total temperature sum available is 124°C higher for warm deme individuals than cold deme individuals (Table 2). Naturally, over the first 14 days of incubation the mean incubation temperatures were also significantly ($F_{1,24}$ = 22.10, P < 0.0001) higher in warm (8.1±0.2°C) than cold streams (6.5±0.2°C). As a consequence, the cold deme individuals will not only, in general, spawn later than warm deme individuals, but the cold deme embryos will have to develop under colder conditions as well. This imposes a double disadvantage for cold deme embryos as they

will have to develop under colder conditions over a shorter period to reach at least a similar size at entrance to the lake.

References

1. Gregersen F, Haugen TO, Vøllestad LA: **Contemporary egg size divergence among sympatric grayling demes with common ancestors.** *Ecol Freshw Fish* 2008, **17:**110–118.
2. Zuur AF, Tuck ID, Bailey N: **Dynamic factor analysis to estimate common trends in fisheries time series.** *Can J Fish Aquat Sci* 2003, **60:**542-552.
3. Kristiansen H, Døving KB: **The migration of spawning stocks of grayling, *Thymallus thymallus,* in Lake Mjøsa, Norway.** *Environ Biol Fishes* 1996, **47:**43-50.
4. Wood SN: **Thin plate regression splines.** *J R Stat Soc, B* 2003, **65:**95-114.
5. Gu C, Wahba G: **Minimizing GCV/GML scores with multiple smoothing parameters via the Newton method.** *Siam J Sci Statist Comp* 1991, **12:**383-398.

Figures

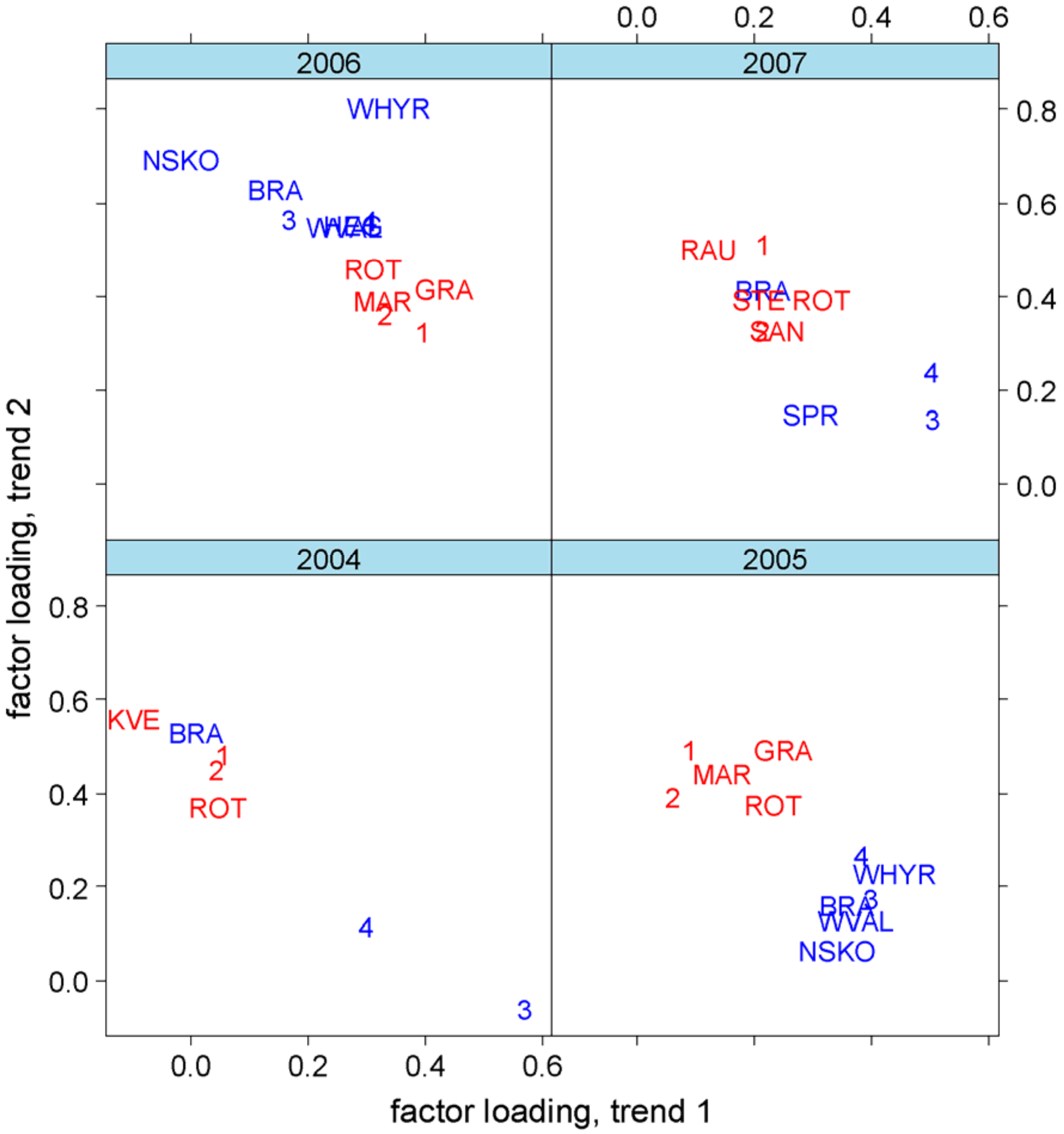

Figure A1. Annual bivariate plots of factor loadings retrieved from the DFA analysis for the two-common-trends models. The pre-defined group assignments of the streams are indicated by different font colouring of the stream abbreviations (blue = cold streams). The four focal demes involved in the current study are numbered according to Figure 1.

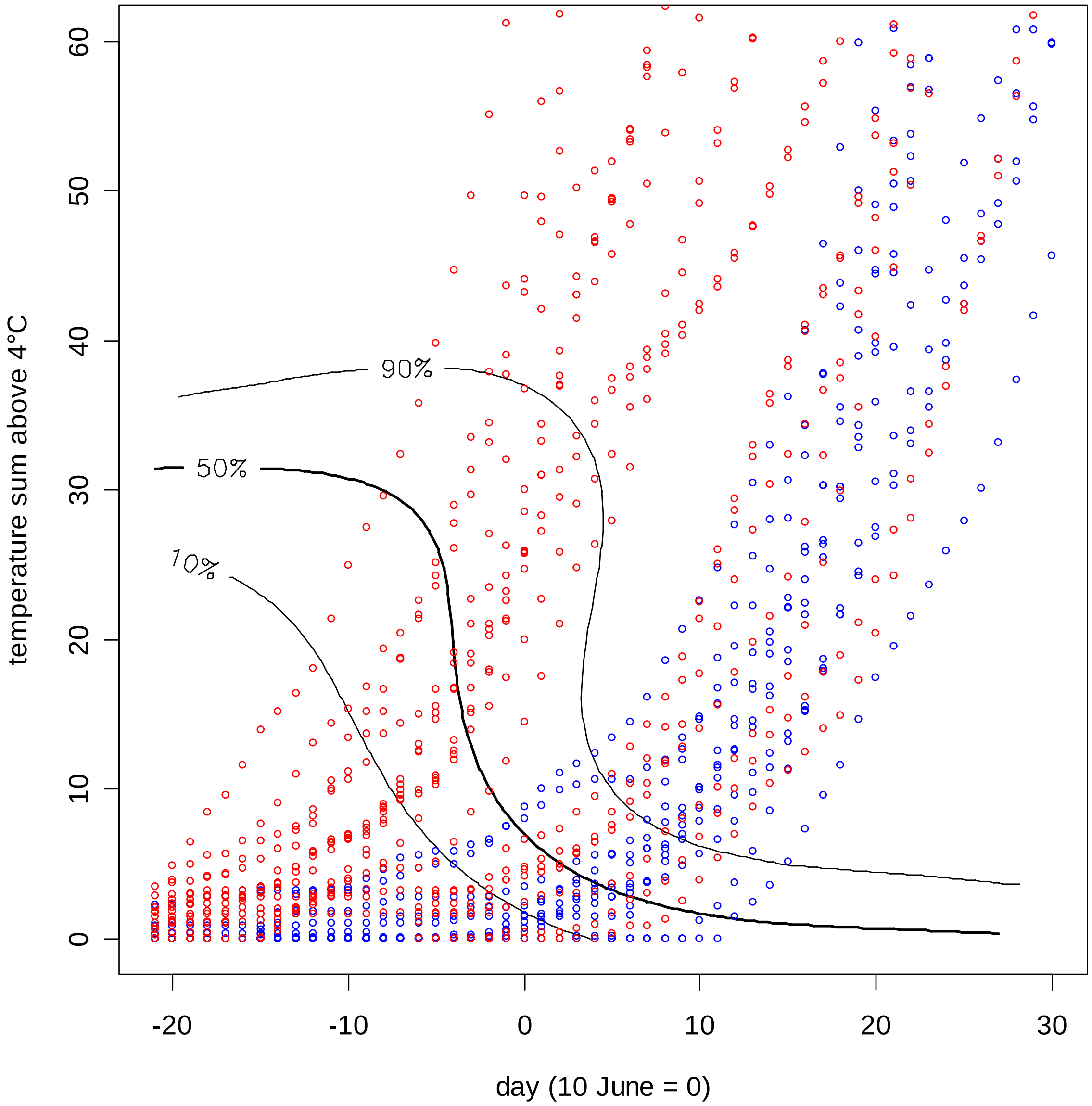

Figure A2. GAM-estimated probability isoclines (black) for the probability of grayling entering the streams for spawning. Blue and red points represent temperature sums based on daily means of measured water temperature in cold and warm streams, respectively.
