# Supplementary material for: Contemporary temperature-driven divergence in a Nordic freshwater fish under conditions commonly thought to hinder adaptation
Source: BMC Evol Biol. 2010 Nov 11;10:350. doi: 10.1186/1471-2148-10-350 (PMC2994878; doi:10.1186/1471-2148-10-350)
Supplement: Additional file 2 — Parameter estimates and model fit statistics for the most supported models fitted to estimate group effects for 11 traits involved in the study (Table A1). [file 1471-2148-10-350-S2.PDF]

Table A1. Parameter estimates and test statistics for the most supported GLM and GAM model structures selected by using Akaike's Information Criterion.

			Parameter estimates				Model fit			
Model type	Response variable	Terms	estimate	s.e.m	*F* or *t*	*P*[1]	R^2_{adj}	df	*F*	*P*[1]
GAM	Notochord length	intercept	9.945	0.048	207.087	***	0.767	5.91, 138.09	81.162	***
		group[w[2]]	2.619	0.092	28.444	***				
		group[w]:deme[2]	0.033	0.170	0.196	0.845				
		group[c[2]]:deme[3]	3.622	0.093	38.856	***				
		group[c]:deme[4]	3.703	0.091	40.534	***				
		s(*DPF*[2])	2.906[3]		134.033	***				
LME (identity)	Muscle fiber size	intercept	-0.884	0.567	-1.558	0.119	0.373	7,28	21.210	***
		NL[2]	0.826	0.313	2.639	0.008				
		deme[2]	7.539	3.964	1.902	0.057				
		deme[3]	1.500	1.605	0.934	0.350				
		deme[4]	-6.335	3.159	-2.005	0.044				
		NL:deme[2]	-0.661	0.395	-1.671	0.094				
		NL:deme[3]	0.058	0.051	1.134	0.256				
		NL:deme[4]	0.544	0.274	1.987	0.046				
		rand(ID)	0.227[4]							
GLM (logit)	Pr(dentary ossified)	intercept	-60.208	17.183	-3.504	**	0.727	4,72	18.996	***
		NL	4.305	1.221	3.526	**				
		group[w]	1.166	0.459	2.540	0.011				
		group[w]:deme[2]	1.669	1.474	1.132	0.257				
		group[c]:deme[3]	-3.247	1.733	-1.874	0.060				
	Pr(oral teeth present)	intercept	-66.333	19.691	-3.369	**	0.757	4,72	19.095	***
		NL	4.741	1.400	3.386	**				
		group[w]	1.341	0.553	2.424	0.015				
		group[w]:deme[2]	0.348	1.524	0.229	0.819				
		group[c]:deme[3]	-5.209	1.978	-2.633	0.008				
	Pr(cleithrum ossified)	intercept	-63.456	18.150	-3.496	**	0.727	4,72	18.996	***
		NL	4.305	1.221	3.525	**				
		group[w]	4.414	1.909	2.312	0.020				
		group[w]:deme[2]	1.669	1.474	1.132	0.257				
		group[c]:deme[4]	3.247	1.733	1.874	0.060				

Pr(operculum ossified)	intercept	-63.456	18.150	-3.496	**	0.727	4,72	18.996	***
	NL	4.305	1.221	3.525	**				
	group[w]	4.414	1.909	2.312	0.020				
	group[w]:deme[2]	1.669	1.474	1.132	0.257				
	group[c]:deme[4]	3.247	1.733	1.874	0.060				
Pr(hypurals>5)	intercept	-94.396	35.599	-2.652	0.008	0.835	4,72	21.689	***
	NL	6.742	2.537	2.658	0.007				
	group[w]	3.833	1.268	3.023	0.002				
	group[w]:deme[2]	3.784	2.169	1.745	0.081				
	group[c]:deme[3]	1.966	2.739	0.718	0.473				
Pr(dorsal vertebral spines)	intercept	-60.208	17.183	-3.504	**	0.727	4,72	18.996	***
	NL	4.305	1.221	3.526	**				
	group[w]	1.166	0.459	2.540	0.011				
	group[w]:deme[2]	1.669	1.474	1.132	0.257				
	group[c]:deme[3]	-3.247	1.733	-1.874	0.060				
Pr(ventral vertebral spines)	intercept	-162.986	80.344	-2.029	0.042	0.845	4,72	22.401	***
	NL	12.091	5.951	2.032	0.042				
	group[w]	1.583	0.964	0.806	0.420				
	group[w]:deme[2]	7.298	4.371	1.670	0.095				
	group[c]:deme[3]	3.542	3.776	0.938	0.348				
Pr(anal fin pterygiophores)	intercept	-30.498	9.101	-3.351	**	0.401	4,72	8.824	***
	NL	2.145	0.638	3.364	**				
	group[w]	-1.904	1.127	-1.690	0.091				
	group[w]:deme[2]	1.740	0.979	1.777	0.075				
	group[c]:deme[3]	-1.601	1.187	-1.349	0.177				
Pr(dorsal fin pterygiophores)	intercept	-84.076	28.204	-2.981	0.002	0.836	4,72	13.198	***
	NL	6.006	2.009	2.990	0.002				
	group[w]	4.222	1.406	3.003	0.002				
	group[w]:deme[2]	1.583	1.763	0.898	0.369				
	group[c]:deme[3]	-1.503	2.312	-0.650	0.515				

[1] ** P<0.001; *** P<0.0001

[2] *NL* = notochord length, *DPF* = days post fertilisation, w = warm streams, c = cold streams

[3] estimated degrees of freedom

[4] fraction of explained variance that is explained by within-individual variation
