# Supplementary figures and images for: Contemporary temperature-driven divergence in a Nordic freshwater fish under conditions commonly thought to hinder adaptation
Source: BMC Evol Biol. 2010 Nov 11;10:350. doi: 10.1186/1471-2148-10-350 (PMC2994878; doi:10.1186/1471-2148-10-350)

$h^2$ 

○ 0.05 △ 0.13 + 0.22 × 0.3 ◇ 0.38 ▼ 0.47 □ 0.55 \* 0.63 ◇ 0.72 ⊕ 0.8

hypurals

muscle fiber area

notochord length

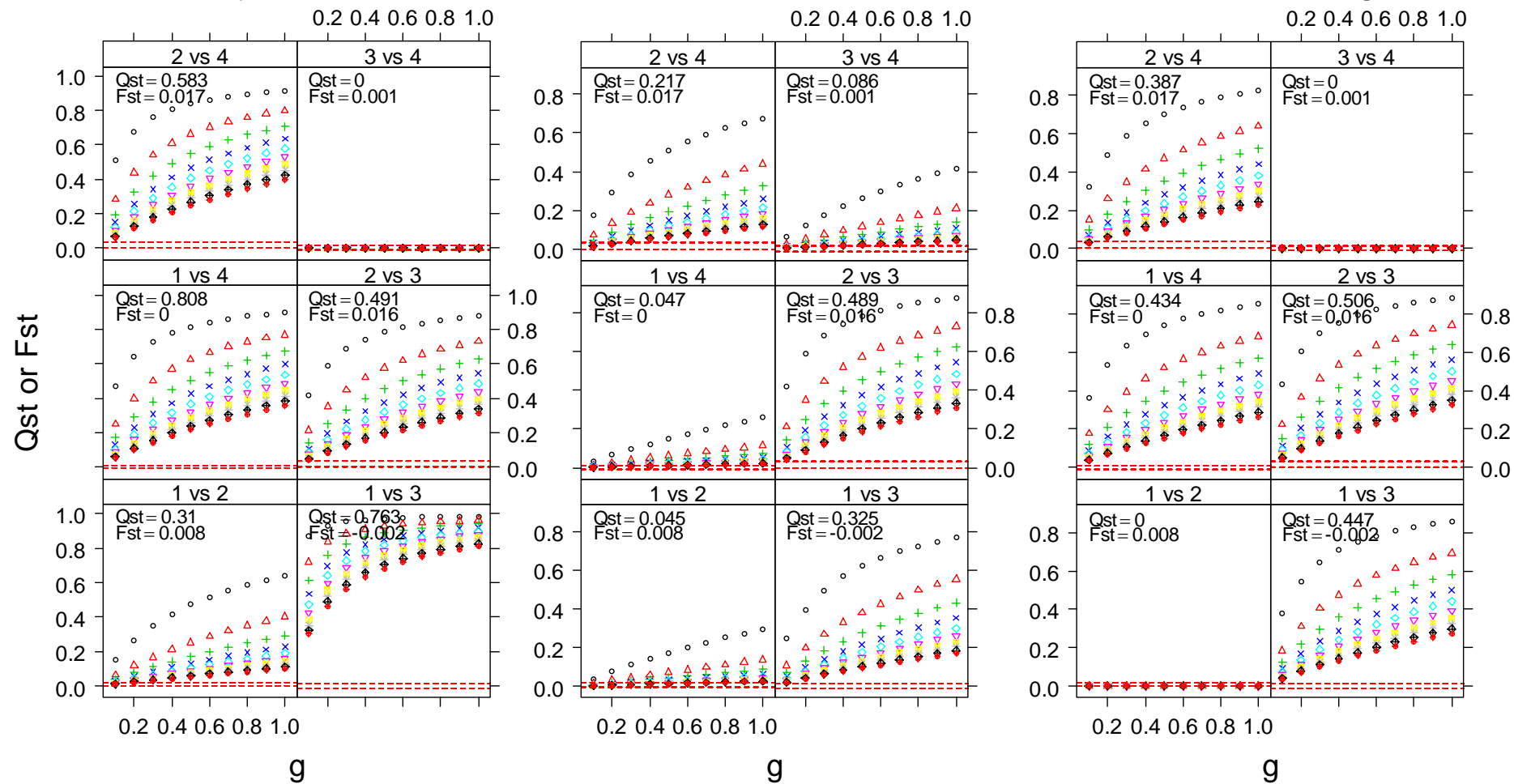

Supplement: Additional file 3 — Qst vs Fst comparisons between pairs of the four demes included in the study (Figure A3). The plots comprise sensitivity plots of QST-values under various g and h2 settings. Left panels show simulations for muscle fiber area, middle panels for notochord length and right panels for the skeletal trait hypurals (as an example of skeletal trait). Horizontal dotted lines indicate the confidence interval for analogous FST-estimates and numbers provided in the figures correspond to mean FST values and expected QST values, respectively. The expected QST corresponds to estimates where g = 0.8 and h2 = 0.3. [file 1471-2148-10-350-S3.PDF]
