# Supplementary material for: Contemporary temperature-driven divergence in a Nordic freshwater fish under conditions commonly thought to hinder adaptation
Source: BMC Evol Biol. 2010 Nov 11;10:350. doi: 10.1186/1471-2148-10-350 (PMC2994878; doi:10.1186/1471-2148-10-350)
Supplement: Additional file 4 — Characteristics of microsatellite loci applied in the study. (Table A2). [file 1471-2148-10-350-S4.PDF]

**Table A2:** Characteristics of microsatellite loci applied in the study. All were run in the same panel; i.e. run in the same capillary.

| **Locus information** | | | **Amplification details** | | | **Genetic diversity** | | |
|---|---|---|---|---|---|---|---|---|
| *Reference*[a] | *Locus*[b] | *Label*[c] | *MP*[d] | *Primer(µM)*[e] | *PCR*[f] | *Na*[g] | *Allele-range*[h] | *Uhe*[i] |
| Genebank: AF151370 | BFRO13 | FAM | MP1 | 0.09 | 58 | 5 | 235-251 | 0.66 |
| (Junge *et al.*, 2009) | 213 | FAM | single | 0.38 | 60 | 11 | 283-327 | 0.78 |
| (Junge *et al.*, 2009) | 414 | FAM | MP2 | *0.20* | 60 | 7 | 389-413 | 0.71 |
| (Junge *et al.*, 2009) | 309 | FAM | MP4 | 0.57 | 59 | 2 | 447-451 | 0.47 |
| (Sušnik *et al.*, 2000) | BFRO10 | VIC | MP1 | 0.08 | 58 | 5 | 96-128 | 0.33 |
| (Sušnik *et al.*, 1999b) | BFRO15 | VIC | MP1 | 0.04 | 58 | 2 | 144-154 | 0.50 |
| (Sušnik *et al.*, 1999b) | BFRO18 | VIC | MP1 | 0.04 | 58 | 5 | 181-195 | 0.59 |
| (Junge *et al.*, 2009) | 207 | VIC | MP1 | 0.10 | 58 | 2 | 216-224 | 0.47 |
| (Sušnik *et al.*, 1999a) | BFRO9 | VIC | MP1 | 0.05 | 58 | 2 | 243-247 | 0.16 |
| (Junge *et al.*, 2009) | 438 | VIC | single | 0.34 | 60 | 10 | 261-297 | 0.79 |
| (Sušnik *et al.*, 2000) | BFRO11 | NED | MP3 | 0.30 | 59 | 2 | 86-102 | 0.43 |
| (Junge *et al.*, 2009) | 313 | NED | MP2 | 0.18 | 60 | 6 | 180-200 | 0.75 |
| (Olsen *et al.*, 1998) | Ogo2 | NED | MP1 | 0.07 | 58 | 4 | 233-245 | 0.63 |
| (Junge *et al.*, 2009) | 433b | NED | MP3 | 0.18 | 59 | 8 | 291-319 | 0.69 |
| (Junge *et al.*, 2009) | 445 | NED | MP4 | 0.13 | 59 | 13 | 374-422 | 0.85 |
| (Junge *et al.*, 2009) | 419a | PET | single | 0.24 | 58 | 2 | 106-110 | 0.29 |
| (Junge *et al.*, 2009) | 415 | PET | MP3 | 0.33 | 59 | 10 | 189-225 | 0.76 |
| (Junge *et al.*, 2009) | 211 | PET | MP2 | 0.25 | 60 | 5 | 232-240 | 0.08 |
| (Junge *et al.*, 2009) | 214 | PET | MP1 | 0.14 | 58 | 3 | 292-313 | 0.53 |

[a]Publication of the locus or Genebank accession number if unpublished.
[b]Common name of the locus
[c]Label, Fluorescent dye used
[d]MP, Multiplex group or single locus PCR amplification
[e]Primer concentration in the PCR-mix
[f]Annealing temperature profile of the PCR program used

[g]Na, Number of alleles found in the locus
[h]*A*-range, Range of the length (bp) of alleles
[i]$H_E$, Unbiased expected heterozygosity in the locus

Marker information and methodological details are described in detail in Junge *et al*. (2009) and Koskinen and Primmer (2001).

**References**

Junge C, Primmer CR, Vøllestad AV, Leder EH (2009). Isolation and characterization of 19 new microsatellites for European grayling, *Thymallus thymallus* (Linnaeus, 1758), and their cross-amplification in four other salmonid species. *Conserv Genet Resour* DOI: 10.1007/s12686-009-9147-z.

Koskinen MT, Primmer CR (2001). High throughput analysis of 17 microsatellite loci in grayling (*Thymallus* spp. Salmonidae). *Conserv Genet* **2**: 173–177.

Olsen JB, Bentzen P, Seeb JE (1998). Characterization of seven microsatellite loci derived from pink salmon. *Mol Ecol* **7**: 1083–1090.

Peakall R, Smouse PE (2006) GENALEX 6: genetic analysis in Excel. Population genetic software for teaching and research. *Mol Ecol Notes* **6**: 288-295.

Sušnik S, Snoj A, Dovc P (1999a). Microsatellites in grayling (*Thymallus thymallus*): Comparison of two geographically remote populations from the Danubian and Adriatic river basin in Slovenia. *Mol Ecol* **8**: 1756–1758.

Sušnik S, Snoj A, Dovc P (1999b). A new set of microsatellite markers for grayling: *BFRO014*, *BFRO015*, *BFRO016*, *BFRO017* and *BFRO018*. *Anim Genet* **30**: 462–478.

Sušnik S, Snoj A, Jesenšek D, Dovc P (2000). Microsatellite DNA markers (BFRO010 and BFRO011) for grayling. *J Anim Sci* **78**: 488–489.
